# Conceptualizing patient participation in psychiatry: A survey describing the voice of patients in outpatient care

**DOI:** 10.1111/hex.13285

**Published:** 2021-05-31

**Authors:** Rikard Wärdig, Fredrik Olofsson, Ann Catrine Eldh

**Affiliations:** ^1^ Department of Health, Medicine and Caring Sciences Linköping University Linköping Sweden; ^2^ Department of Psychiatry in Norrköping and Department of Biomedical and Clinical Sciences Linköping University Linköping Sweden; ^3^ Department of Public Health and Caring Sciences Uppsala University Uppsala Sweden

**Keywords:** concept, outpatient, patient participation, person‐centred care, psychiatry, questionnaire, survey

## Abstract

**Background:**

While increasingly discussed in somatic care, the concept of patient participation remains unsettled in psychiatric care, potentially impeding person‐centred experiences.

**Objective:**

To describe outpatient psychiatric care patients’ conceptualization of patient participation.

**Design:**

An exploratory survey.

**Setting and participants:**

Patients in four psychiatric outpatient care units.

**Variables:**

Patients conceptualized patient participation by completing a semi‐structured questionnaire, including optional attributes and free text. Data were analysed using statistics for ordinal data and content analysis for free text.

**Results:**

In total, 137 patients (69% of potential respondents) completed the questionnaire. The discrete items were favoured for conceptualizing patient participation, indicating a primary connotation that participation means being listened to, being in a reciprocal dialogue, learning about one's health care and managing one's symptoms. Additional free‐text responses acknowledged the attributes previously recognized, and provided supplementary notions, including that patient participation is about mutual respect and shared trust.

**Discussion:**

What patient participation is and how it can be facilitated needs to be agreed in order to enable preference‐based patient participation. Patients in outpatient psychiatric care conceptualize participation in terms of both sharing of and sharing in, including taking part in joint and solo activities, such as a reciprocal dialogue and managing symptoms by yourself.

**Conclusion:**

While being a patient in psychiatric care has been associated with a lack of voice, an increased understanding of patient participation enables person‐centred care, with the benefits of collaboration, co‐production and enhanced quality of care.

**Patient contribution:**

Patients provided their conceptualization of patient participation in accordance with their lived experience.

## 1 INTRODUCTION

Patient participation is a common standard in modern health care,^1^ although it is sometimes phrased as patient, client, or user engagement or involvement.^2^ Although there is a similarity between the word participation, engagement and involvement, only participation originates from and primarily emphasizes ‘sharing’.^3^ Perhaps as a result, and recognizing the necessity for a mutual sharing of the individual patient's lived experience and preferences, and the knowledge and experience of health‐care staff, patient participation is a term that is frequently applied in health‐care legislation and policies.^4^ Nevertheless, from time to time patient participation has been interpreted in a reduced sense, essentially addressing patient engagement in decision making.^5^ Such limited features do not correspond to the richness and breadth of the concept^6^, ^7^, ^8^, ^9^ and may hinder the realization of patient participation in health‐care interactions.^10^, ^11^ Knowing that, to people enacting a patient role, patient participation means shared information and mutual knowledge exchange, being engaged in plans and decisions, as well as in self‐care actions, and in performing certain proportion of one's treatment,^12^, ^13^, ^14^ the employment of a wider notion of participation is crucial.^15^

Compared with somatic care, there are fewer studies conceptualizing patient participation from a patient perspective in psychiatric care,^16^, ^17^, ^18^ although a recognition of the patient's experience is central to person‐centred psychiatry.^19^ Health professionals are at times the main source when it comes to defining core concepts such as patient participation,^18^ even though nurses have emphasized the necessity to recognize the individual patient and his or her needs in psychiatry.^20^ Yet, clients have had limited power to speak out or articulate their needs and preferences,^18^ leaving nurses in psychiatric care with difficulties arriving at a common idea of patient participation.^21^, ^22^ Perhaps this is a result of there being no, or limited, support to conceptually guide nurses and other professionals in everyday psychiatric care encounters.^23^ Although many psychiatric conditions can limit one's ability to make an informed choice vis‐à‐vis treatments,^24^ nurses are aware that participation can have different connotations for patients than what they as professionals suppose.^25^

Low levels of patient participation affect patient satisfaction negatively and have an adverse impact on quality of care,^1^ but without a shared understanding of the connotations of patient participation, the provision of person‐centred patient participation is hampered.^26^ The individual's preferences are central to person‐centred care, comprising a recognition of experiences, needs and resources of clients in mental health care.^27^ The aim of this study was to describe outpatient psychiatric care patients’ conceptualization of patient participation.

## 2 METHODS

### 2.1 Design

The study was conducted using an exploratory design^28^ to develop an understanding of a particular phenomenon.^29^

### 2.2 Setting

Four outpatient psychiatry clinics for adults in Sweden were enrolled in the study. The region includes urban and rural communities, towns and cities, and the units were run by the region or procured by the same to provide outpatient services. The head of department at each clinic gave written approval, after receiving information about the study purpose, procedures and ethical considerations.

### 2.3 Procedure

After data collection authorization, the staff at each clinic were informed about the study by author FO and asked to assist with the distribution of patient information and questionnaires, and to store sealed envelopes with responses. A total of 200 questionnaires were envisaged in order to achieve at least 120 responses, accounting for a rule of thumb that 10 respondents per item are preferred. Thus, each clinic was given 50 questionnaires to distribute and retrieve.

Staff were instructed to inform patients about the study and its purpose, and to emphasize that it was about patients’ perceptions of the concept of patient participation (rather than an evaluation of whether or not patient participation had occurred in their health‐care contact).

Patients scheduled for outpatient visits between mid‐December 2018 and March 2019 were consecutively provided with the verbal information by the member of staff registering their arrival at the clinic and asked whether they were willing to consider the study. Exclusions were made for patients who could not respond without assistance, including those not speaking Swedish or suffering a cognitive impairment. Patients who agreed received the information letter, the patient participation questionnaire and a short survey regarding demographics, along with an envelope to dispatch the reply to the research team. The written information addressed the study's purpose and confidentiality issues, clarifying that a response was voluntary but would be considered informed consent. The patient was also informed in writing that he or she could drop the questionnaire in the waiting area without replying or place it in the envelope, reinforcing the voluntariness of participation.

To guarantee confidentiality, no registration was made as to who agreed to receive the information‐questionnaire package and who declined. Rather, as the questionnaires were collated by the researchers, they were coded consecutively in order to maintain traceability in the recording of data and the ability to member‐check accurate data registrations.

### 2.4 Data collection

The patients were asked to anonymously conceptualize patient participation, using a questionnaire of the Patient Preferences for Patient Participation family, the 4Ps.^30^ It includes a single query, which asks the respondent to describe ‘what patient participation means to me’. The patient can choose to employ discrete attributes and/or free text. While the 12 listed attributes draw upon previous studies, including concept analyses, semantics, legislation and other policies, in addition to scientific studies, including patients’ conceptualizations of the somatic care context, none of this is revealed to the respondent.^30^, ^31^ Rather, the responding patient is instructed to tick the or those attribute(s)—if any—which represent patient participation to him or her, and to use the free‐text spaces to impart any other or additional descriptions of patient participation.

### 2.5 Data analyses

The quantitative data collected were registered in Microsoft Excel. As it consisted of whether a suggested attribute was ticked or not and the demographics, data were nominal and ordinal, respectively, and analysed for incidence and frequency.^28^

The qualitative data, consisting of the free‐text responses, were registered as text, also in Microsoft Excel, and later stored in a separate file and subjected to content analysis^32^:

- Firstly, all free‐text responses were considered as one and read inductively to attain a sense of overall understanding.
- Secondly, each free‐text response was deductively analysed to identify whether (a) associated with the listed attributes, considering all discrete items and the ones the particular respondent had employed to conceptualize patient participation, or (b) comprised other features.
- To conclude, the type (b) free‐text responses were compared with the conceptual, semantic or scientific boundaries of patient participation in a deductive phase.^12^, ^15^, ^33^, ^34^ Those that corresponded were inductively analysed for core content, while those that did not were further (deductively) considered in relation to other health‐care concepts associated with quality of care.^35^

### 2.6 Ethics

The study was reviewed by the Ethical Review Board in Linköping, Sweden, and found not to fall under the research ethics legislation (ID 2018/489‐31). The board's advice to revise the letter of information to patients was considered, and an adjustment omitted advanced terms, thus simplifying the letter.

## 3 RESULTS

To set the study's context, the respondents’ demographics are first presented, before their conceptualizations of patient participation by means of attributes listed and free texts, respectively.

### 3.1 Response rate and demographics

A total of 137 questionnaires were completed and returned. Only one questionnaire was submitted with no content, generating a response rate of 69%. As for internal response dropout, one patient did not state his or her age.

A majority of the respondents were women (77%) with a mean age of 37 years (19‐66 years). The largest group had been patients in psychiatric outpatient care for 1‐5 years (n = 48, or 35% of the respondents), followed by patients who had been in contact with psychiatric outpatient care for 10 years or more (n = 41, 30%). All demographic details are shown in Table 1.

**TABLE 1:** Demographics of respondents

Variable	n = 137
Gender	No. (%)
Women	106 (77)
Men	28 (20)
Non‐binary persons	3 (2)

Highest level of education	No. (%)
Elementary school	23 (17)
High school	72 (53)
University or university college	41 (30)
Missing	1 (<1)

Time as patient in psychiatric care	Frequency (%)
<1 mo	2 (1)
1‐5 mo	9 (7)
6‐12 mo	17 (12)
1‐5 y	48 (35)
6‐10 y	20 (15)
>10 y	41 (30)

### 3.2 Patients’ conceptualization of participation by means of proposed attributes

As shown in Table 2, all 12 discrete attributes were used by respondents to indicate what patient participation signifies. Furthermore, the attributes were not indicated by their proposed order to conceptualize participation ‘as it means to me’, and there was a variance in how many patients had considered each of the discrete attributes. The attribute that most, 90% (n = 123), used to signify patient participation was ‘being listened to by the health‐care staff’ (listed as discrete attribute number 1 in the questionnaire). The second most frequent attribute was ‘having conditions for reciprocal communication’ (submitted as number 3 in the order of discrete items), followed by ‘having explanations of what is [being] done for oneself’ (listed as number 6 in the discrete items). The attribute ‘performing self‐care, such as adjusting diet’ (the last out of 12 discrete items) was the one attribute ticked by the fewest respondents to describe patient participation, although 52% (n = 71) employed it for their conceptualization.

**TABLE 2:** Patients’ conceptualizations of patient participation using the proposed attributes

Attributes included in the questionnaire, in order	n = 137 (%)
Being listened to by the health‐care staff	123 (90)
One's experience being recognised	90 (66)
Having conditions for reciprocal communication	117 (85)
Sharing one's symptoms/issues	110 (80)
Having explanations for one's symptoms/issues	104 (76)
Having explanations for what is done for oneself	116 (85)
Learning about plans	108 (79)
Partnering in planning of care/treatment	108 (79)
Phrasing one's own goals	85 (62)
Being able to manage one's symptoms/issues	113 (82)
Managing health‐care interventions oneself, such as medication	84 (61)
Performing self‐care, such as adjusting diet	71 (52)

### 3.3 Patients’ conceptualization of participation by means of free‐text responses

An additional 36 free‐text responses were submitted for what patient participation is, provided by 24 of the participants (18%). These represented a similar gender balance to the respondents in general: 18 women (75%) and four men (17%), along with two of the three non‐binary persons.

Most free‐text responses corresponded to the attributes of patient participation distinguished in the questionnaire, but provided additional nuances, as illustrated in Table 3. For example, the attribute ‘being listened to by the health‐care staff’ also comprised a sense of recognition and approval of one's experience; the attribute ‘having conditions for reciprocal communication’ was also emphasized as involving a sense of mutual trust. Furthermore, ‘having explanations for one's symptoms/issues’ included access to research information, whereas both attributes connoting health and self‐care management mirrored an additional sense of self‐confidence.

**TABLE 3:** Outcome of free‐text response analysis, in relation to current attributes and additional concepts

Outcome of analysis	Correspondence with attributes conceptualizing patient participation	Correspondence with additional health‐care concept
Being recognized and taken seriously (n = 7)	Being listened to by the health‐care staff	
Having a good encounter characterized by dialogue and trust (n = 9)	Having conditions for reciprocal communication	
Having access to research information (n = 1)	Having explanations for one's symptoms/issues	
Being confident in terms of medication management (n = 1)	Managing health‐care interventions oneself	
Being confident in terms of self‐care (n = 1)	Performing self‐care	
Having enhanced availability of health‐care services		Health‐care access
Having access to a multi‐professional team		Multi‐professional teams
Better continuity of care		Continuity of care

In addition, two free‐text responses emphasized that the listed attributes echoed the conceptualization of patient participation. The responses that did not semantically fit patient participation illuminated the need for enhanced continuity of care and access to multi‐professional teams in psychiatric care, as shown in Table 3.

## 4 DISCUSSION

The World Medical Association's ethical standards emphasize the necessity of including vulnerable groups in research,^36^ indicating the importance of recognizing patients with psychiatric experience in defining concepts that can facilitate person‐centred patient participation. Previous research that has included the user experience of his or her role as a patient in different psychiatric contexts employs a variety of labels for this: for example, Curwen et al^37^ describe the benefits of ‘people participation’, indicating both increased self‐confidence and the development of personal skills. Armstrong et al^38^ on the other hand, describe ‘patient involvement’ with patients as part of a team in clinical development. Deegan suggests that, rather than arguing over which is the correct term, a further emphasis on reciprocal liaisons is needed within the psychiatric context; personhood must be recognized above the psychiatric diagnosis, acknowledging that recovery from mental illness also includes liberation from stigma. Consequently, participation is enacted as the regaining of a sense of power and sovereignty over one's own life, as well as the right to choose.^39^

A previous focus on decision making as a primary route to substantiate participation has more recently been replaced by policies assisting the enactment of person‐centred care, including the conditions to partake in alignment with one's preferences. Health care should be framed and executed in collaboration, incorporating the individual's preferences, with patient participation reinforcing the person‐centred perspective.^1^ Yet, previous studies have shown that the conditions are far from optimal for preference‐based patient participation and that further efforts are needed to create an enhanced and person‐centred standard.^31^, ^34^, ^40^, ^41^

If professionals continue to commonly associate patient participation in psychiatric care with decision making,^42^, ^43^ it excludes a full understanding and provision of preference‐based patient participation and hampers the engagement of mental care clients who are not used to making decisions.^9^ Fortunately, there is growing understanding that decision making should be characterized by the hallmarks of a shared decision,^44^ that is a process arriving at a mutual understanding of what is opportune for the individual—recognizing the patient's experiences and preferences, in addition to the professional's knowledge and understanding.^4^ In psychiatric care, this may be more commonly known as ‘patient involvement’, illustrating the ambition to advance person‐centred services.^45^, ^46^ Yet, even though involvement and participation are similar concepts, participation in particular conveys the concept of the sharing of knowledge and respect,^47^ calling for investigations to address it further.

The attributes used by most clients engaged in this study to conceptualize participation were ‘being listened to by the health‐care staff’ and ‘having conditions for reciprocal communication’. This indicates that professionals in psychiatric care can provide for patient participation without surrendering their responsibility for evidence‐based care. While communication is central to patient participation, an individual approach is especially important if a patient suffers from an inability to make rational decisions,^48^ and facilitating patients’ need to be listened to also contributes to a shared understanding.^49^ In addition, the free‐text responses highlighted the nuances of a mutual respect and shared trust, further conceptualizing the ‘sharing of’ and ‘sharing in’ perspectives of patient participation in a psychiatric context. Thus, outpatient psychiatric care clients conceptualize participation much like patients in somatic care, indicating a shared understanding, which is something that humans tend to strive for in the sense of belonging and co‐creating a common conceptual lifeworld.^50^

Of the study participants, 80% or more chose to employ five of the proposed attributes to conceptualize patient participation: being listened to, having conditions for reciprocal communication, sharing symptoms and issues, having explanations about what is being done for me, and being able to manage symptoms and issues. The common denominator for four of these is that they are about being heard and recognized. While fundamentally important no matter who you are, this may be particularly important when suffering from mental illness, which creates doubt in one's own abilities.^51^ Many people with mental illness assume a sense of poor self‐esteem,^52^ which is especially correlated with depression.^53^ People who experience difficulties in maintaining their preferred identity may choose to exclude themselves from social interactions.^54^ This requires all health care, and psychiatric services in particular, to recognize the significance of a dialogue that comprises a genuine consideration for the person, and acknowledging his or her say.^55^ Deegan, sharing a personal experience perspective on mental illness and recovery, emphasizes that people are not passive recipients of interventions and remedies, but agents in a transition towards renewed self‐esteem and meaning beyond the disability.^39^

In this study, patients less often defined patient participation as ‘to perform self‐care’. Psychiatric symptoms have been shown to constitute barriers to self‐care,^56^ even if the patient's insight into their disease promotes the ability to perform self‐care.^57^ In the questionnaire used in this study, this attribute was exemplified by ‘…, eg to adjust my diet’, which may have hampered a broader conceptualization; for patients with psychiatric disorders, there are major challenges involved in changing lifestyle.^58^ The concept of self‐care has been used to only a limited extent in relation to mental health,^59^ being more often addressed as self‐management and recovery.^60^ Further research is suggested into how to support self‐care in psychiatric outpatient care, particularly investigating the patient perspective. Yet, recognizing the patient's experience is vital for person‐centred care, because service users are stakeholders with a unique insight into what really does constitute quality of care.^61^

### 4.1 Strengths and limitations

A translation of our study findings to psychiatric care in general should be conducted with some vigilance: although the respondents represent a fairly typical profile of the larger group of people who attend outpatient psychiatric care, including a majority being women,^62^ the responses stem from people who volunteered to take part.^63^ Yet, since the study neither evaluated the respondents’ experiences of patient participation, nor asked for a rating of the importance of the attributes conceptualizing patient participation, further investigations of such aspects are suggested.

The inclusion of discrete attributes could constitute a limitation because they may have impeded a further conceptualization of patient participation. However, since the results were not linked to the order in which the attributes were posted in the questionnaire, we suggest the patients considered and deliberately used the items for conceptualizing patient participation. This would demonstrate that the findings represent a more general understanding of the phenomenon from a lived experience perspective, similar to those captured in other health‐care services for adults.^64^ In addition, the free‐text responses indicated that the listed attributes provided both for endorsements and for additional nuances beyond what was previously known regarding patient participation. Some of the additional perspectives presented were found to correspond to further aspects of quality of care, rather than patient participation in particular; thus, a lay word such as patient participation can, in spite of its particular connotation, be associated with other positive attributes of care.

## 5 CONCLUSION

What patient participation is and how it can be supported has to be agreed between the patient and the health‐care professionals on his or her team in order to facilitate patient participation consistent with the individual's preferences. This study demonstrates that both sharing of experiences and sharing in activities are favoured when patients in outpatient psychiatric care conceptualize patient participation. Patient participation signifying a recognition of the patient's voice imply reciprocal interactions in mental health care where health‐care professionals and service users engage to facilitate preference‐based patient participation. The prospect of a means such as the 4Ps tool to facilitate open psychiatric care dialogues requires further research, incorporating strategies to implement person‐centred patient participation in mental health care.

## CONFLICT OF INTEREST

The authors declare that they have no competing interests. The 4Ps is a non‐commercial product protected by copyright and available from the last author by agreement.

## AUTHORS’ CONTRIBUTIONS

ACE planned the study. FO made and secured all the contacts with the study units, distributed and collected the questionnaires, registered and analysed the data and made a tentative report. ACE and RW drafted the paper, in collaboration with FO. All authors have been engaged in the writing and revising of the paper and have agreed to the final version of the manuscript.

## Data Availability

Data are available on request due to privacy/ethical restrictions.
